# Steroid-Refractory Acute GVHD: Predictors and Outcomes

**DOI:** 10.1155/2011/601953

**Published:** 2011-11-03

**Authors:** Jason R. Westin, Rima M. Saliba, Marcos De Lima, Amin Alousi, Chitra Hosing, Muzaffar H. Qazilbash, Issa F. Khouri, Elizabeth J. Shpall, Paolo Anderlini, Gabriela Rondon, Borje S. Andersson, Richard Champlin, Daniel R. Couriel

**Affiliations:** ^1^Stem Cell Transplantation & Cellular Therapy, M.D. Anderson Cancer Center, Houston, TX 77030, USA; ^2^Blood and Marrow Transplant Program, University of Michigan Comprehensive Cancer Center, Ann Arbor, MI 48109, USA

## Abstract

Patients with steroid-resistant acute graft versus host disease (aGVHD) have a dismal prognosis, with mortality rates in excess of 90%. We sought to identify a subgroup of patients less likely to benefit from initial therapy with corticosteroids as well as the impact of response on day 14 on outcome. Retrospective evaluation was performed of patients with biopsy-proven aGVHD treated with corticosteroids after allogeneic HSCT at M.D. Anderson Cancer Center from 1998 through 2002 (*N* = 287). Overall response to first-line therapy on day 14 was 56%. Grade III-IV aGVHD and hyperacute GVHD were the most significant factors predicting failure. Patients who fail to respond to steroids by day 14 should be considered for clinical trials. Severity of aGVHD, hyperacute GVHD, and sex mismatch could be integrated into prognostic scoring systems which may allow for pretreatment identification of patients unlikely to benefit from standard therapy with corticosteroids.

## 1. Introduction

Acute graft-versus-host disease (aGVHD) remains one of the major limiting factors in successful allogeneic hematopoietic stem cell transplantation (HSCT) [1, 2]. Standard treatment for aGVHD consists of corticosteroids, although there is a lack of consensus over optimal dosing and schedule [3]. Response to corticosteroids is seen in approximately 50% of patients [2], and those who fail initial therapy have mortality rates as high as 95% [4].

Response to first-line therapy as a predictor of outcome has been systematically assessed in multiple recent studies [5–7] which concurred that both day 14 and day 28 responses were highly correlated with outcome and each other. However, day 28 response was the strongest predictor statistically. These findings are in line with a consensus statement endorsing day 28 response as the optimal endpoint in aGVHD treatment trials [3].

There are currently no established prognostic models or biomarkers to assist in the identification of patients at high risk of failing first-line therapy. We have previously described the hyperacute presentation, defined as that occurring within 14 days after transplantation irrespective of engraftment, as associated with inferior response rate and survival outcomes [8]. In the current study, we systematically evaluate patient, transplant, and aGVHD characteristics as predictors for failure to respond to initial therapy with corticosteroids in a large patient population treated with standard first-line therapy. We specifically evaluated the effect of these risk factors on response to initial therapy with corticosteroids on day 14 and the impact of response on nonrelapse mortality (NRM).

## 2. Materials and Methods

### 2.1. Patients

We studied all consecutive patients who underwent an allogeneic HSCT as part of prospective clinical trials at the University of Texas M.D. Anderson Cancer Center between January 1998 and September 2002. Patients who received umbilical cord blood transplantation or T-cell depleted graft were excluded from this study. HLA typing was intermediate resolution for Class I antigens (HLA-A and B) until 2001 (high resolution thereafter for matched unrelated donor grafts) and high-resolution for HLA-DRB1. If a patient received more than one allogeneic transplant during the study period, only the initial transplant was utilized for the statistical analysis, and further data was censored at the time of subsequent transplantation. Patients who had primary graft failure were not eligible for inclusion in the study. 

All patients were treated on Institutional Review Board approved research protocols at the M.D. Anderson Cancer Center. Patients signed informed consent in accordance with the Declaration of Helsinki. Demographic and clinical data were retrieved from the Department of Stem Cell Transplantation and Cellular Therapy electronic database, which is prospectively updated according to standardized data entry criteria and from the electronic medical records. A retrospective chart review protocol was approved by the institutional IRB for the current analysis.

### 2.2. Conditioning Regimens and GVHD Prophylaxis

Conditioning regimens are listed in Table 1. Myeloablative regimens were expected to result in profound pancytopenia for greater than 28 days, and hematopoietic recovery was completely donor-derived. Reduced-intensity regimens were defined as those in which recipient hematopoietic recovery was expected to occur within 28 days without transplantation and, after transplantation, chimerism could be documented in most patients [9, 10].

### 2.3. Engraftment

Date of engraftment was defined as the first of 3 consecutive days in which the patient had an absolute neutrophil count of 0.5 × 10^9^/L or greater. Failure to engraft by day 30 was considered primary graft failure. Secondary graft failure is defined as failure to sustain an absolute neutrophil count 0.5 × 10^9^/L or greater after attainment of primary engraftment.

### 2.4. Assessment of aGVHD

Diagnosis of aGVHD involved clinical features, positive biopsy results from at least one involved organ, and exclusion of other causes of rash, diarrhea, and liver function abnormalities. Patients without biopsy-proven GVHD were excluded from the analysis (*n* = 55). The staging and grading of aGVHD were performed using the modified Glucksberg consensus criteria and occurred at the time of initiation of treatment [11]. We defined hyperacute GVHD as aGVHD occurring within the first 14 days after HSCT as previously published [8]. Mild-to-moderate aGVHD includes patients with grade I and II disease, while grades III and IV were considered severe.

### 2.5. Evaluation of Response to Therapy

Each organ system was prospectively and retrospectively assessed for response to therapy. Complete responses (CR) and partial responses (PR) were assessed 14 days after the initiation of therapy [2]. A CR was defined as the complete resolution of all manifestations of aGVHD. A PR was a decrease in organ stage by 1. Progressive disease (PD) was defined as an increase in organ stage by 1 and was evaluated 48 hours (gastrointestinal (GI) and liver) or 72 hours (skin) after the initiation of corticosteroids. Patients were considered nonresponders (NRs) in the absence of CR, PR, or PD 7 days after the initiation of corticosteroids for skin GVHD or 72 hours after its initiation for GI and liver GVHD. Overall response integrated the responses at all sites (skin, GI, and liver). An overall CR was defined as the resolution of GVHD in all evaluable organs for a minimum duration of 14 days. Overall PR was any improvement in at least one evaluable organ without deterioration of others. Overall PD was deterioration in at least one evaluable organ without improvement of the others. NR was the absence of any change or any situation other than CR, PR, or PD. Thus, steroid refractory patients were those in the NR and PD categories.

### 2.6. Statistical Methods

Analysis was performed on the basis of outcomes documented by July 2007. Patients who experienced secondary graft failure after neutrophil engraftment were censored at the time of the secondary graft failure. Predictors of response to first line therapy in patients diagnosed with grade I–IV aGVHD were assessed on univariate and multivariate analysis using logistic regression analysis. Factors evaluated included grade of aGVHD at the time of initiation of systemic therapy, time of onset of aGVHD, patient age, donor/patient gender, donor type, cell type, intensity of conditioning regimen, underlying malignancy, disease status at transplantation, number of prior chemotherapy regimens received, infused dose of CD34+ cells, and the steroid dose at the initiation of systemic therapy. Predictors of NRM were assessed on univariate and multivariate analysis using Cox's proportional hazards model [12]. The cumulative incidence of NRM and chronic GVHD were estimated by the cumulative incidence method [13] considering death due to persistence or recurrence of underlying malignancy and death before the development of chronic GVHD as competing risks, respectively. Actuarial survival was estimated by the Kaplan-Meier method. All outcomes were estimated since the initiation of first-line therapy. Factors significant at the 0.1 level on univariate analysis were considered for multivariate analyses using backward elimination. Two-sided *P* values less than  .05 were considered significant. Analysis was performed using STATA 7.0 (Stata, College Station, TX).

## 3. Results

### 3.1. Demographics and Incidence of Acute GVHD

A total of 818 patients received allogeneic HSCT transplantation during the study period including 287 who met the inclusion criteria for the current analysis. Patient characteristics are listed in Table 1. Of the 287 patients, 198 (69%) developed grade II–IV aGVHD. Nearly all patients received tacrolimus (98%, *n* = 283) or tacrolimus and methotrexate (*n* = 276, 96%) starting on day −2, targeting blood levels of 5–15 ng/dL. Patients were started on methylprednisolone at 2 mg/kg daily at the time of onset of aGVHD. A subset of patients with grade I-II aGVHD received less than 2 mg/kg of daily methylprednisolone (*n* = 24, median 1 mg/kg, range 0.3–1.5 mg/kg) as initial therapy. Pentostatin (*n* = 10, 3%) or corticosteroids (*n* = 3, 1%) were added to the GVHD prophylaxis regimen as part of studies involving unrelated donor or HLA-mismatched transplants, and antithymocyte globulin was given as part of the conditioning regimen in 79 (29.6%) patients. 

Seventy-six patients (26%) developed aGVHD prior to day 14 and were categorized as hyperacute GVHD, of which 13 were categorized as severe. Seventy-three patients (25%) were sex mismatched (female donor and male recipient), with 16 categorized as severe. In about half of the patients (*n* = 132, 46%), skin was the only organ involved.

### 3.2. Frontline Treatment of aGVHD

Patients with grade I–IV aGVHD were started on methylprednisolone (MP) according to institutional guidelines (Table 2). Tacrolimus was continued at blood levels between 5 and 15 ng/dL. A total of 27 patients (9%) received one or more additional immunosuppressants for frontline treatment of aGVHD, including infliximab (*n* = 17, 6%), daclizumab (*n* = 5, 2%), pentostatin (*n* = 3, 1%), and basiliximab (*n* = 4, 1%).

### 3.3. Response to First-Line Therapy for Acute GVHD

Of the 287 patients, 161 (56%) achieved a CR or PR at two weeks after the initiation of first line therapy (Table 2). Time to development of aGVHD was assessed by quartiles, and day 14 corresponded to the 25th percentile of the distribution our study population. The majority of patients who failed first-line corticosteroids were subsequently treated on protocols with various investigational agents. 

On univariate analysis, overall grade of aGVHD at the time of initiation of systemic therapy was the strongest predictor of response (CR or PR). A significantly higher proportion of patients with mild-to-moderate aGVHD responded to first-line therapy than those with severe aGVHD (60% versus 39%, *P* = 0.003). The proportion of responders was similar in patients with grade I (65%) and II (57%) aGVHD (*P* = 0.2) in the mild-to-moderate group, and in those with grade III (39%) and IV (33%) aGVHD (*P* = 0.7) in the severe group. Response was also significantly lower for patients who were diagnosed with hyperacute GVHD compared with those whose aGVHD was diagnosed later (42% versus 61%, *P* = 0.007). The lower response rate for patients with hyperacute GVHD was seen in patients with mild-to-moderate aGVHD (48% versus 65%, *P* = 0.03) as well as those severe aGVHD (15% versus 45%, *P* = 0.05). Conversely, sex mismatch (female donor and male recipient) was associated with significantly lower response in patients with grade III-IV (CR/PR 12% versus 49%,  *P* = 0.01) but not in patients with grade I-II (CR/PR 61% versus 60%,  *P* = 0.9). There was also a trend for higher response in patients who had active disease at transplant, and those who received a grafted from a matched donor. 

Factors that were significant (*P* < 0.05) or marginally significant (*P* < 0.1) on univariate analysis were assessed in multivariate analysis including the severity of aGVHD at the initiation of therapy (grade I-II versus grade III-IV), time of onset of aGVHD (hyperacute versus other), donor type, and disease status at the time of transplantation. Sex mismatch in the severe aGVHD group was not considered in multivariate analysis because the estimate was based on a very small number of patients. Among these factors, only severity and time of onset of aGVHD remained significant on multivariate analysis. 

To quantify the independent effects of severity and time of onset of aGVHD, the multivariate model was constructed based on four mutually exclusive groups of patients depending on the severity and the time of onset of aGVHD (Table 3). Patients who had mild-to-moderate aGVHD and were diagnosed with aGVHD beyond day 14 from transplantation (*n* = 170) had a significantly higher response rate (65%), followed in decreasing order of response by those who had mild-to-moderate hyperacute aGVHD (*n* = 60, 48%, *P* = 0.03), severe aGVHD diagnosed after day +14 (*n* = 44, 45%, *P* = 0.02), and severe hyperacute aGVHD (*n* = 13, 15%, *P* = 0.003). This grouping of patients will be used for further evaluation of the independent effect of severity and time of onset of aGVHD on survival. 

### 3.4. Overall Survival and Nonrelapse Mortality

**Figure 1:**
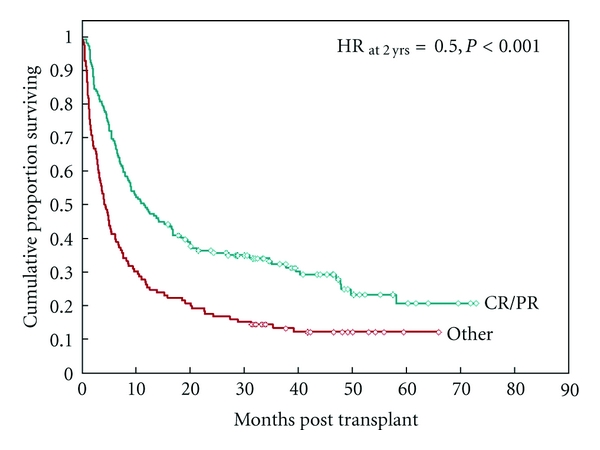
Overall survival in steroid responsive and refractory aGVHD patients.

The median followup among survivors was 42 months (range 16–73). Actuarial survival was 41% in responders and 22% in nonresponders at 18 months (*P* < 0.001), and 36% versus 17% (*P* < 0.001) at 2 years, respectively (Figure 1). Similarly, patients who failed to respond to first-line therapy suffered higher NRM at 18 months (63% versus 34%, *P* < 0.001) and 2 years (65% versus 35%, *P* < 0.001) since initiation of therapy. Causes of death according to response to first line therapy are presented in Table 4. Death was attributed to aGVHD in 35% of cases in nonresponders versus 3% of responders (*P* = 0.013). The rate of GVHD mortality (including both acute and chronic) was significantly lower in the responding group (HR = 0.2, *P* < 0.001). 

### 3.5. Predictors of Nonrelapse Mortality

On univariate analysis, failure to respond was the most significant predictor of NRM at two years after initiation of first-line therapy (HR = 0.4, *P* < 0.001). This effect was consistent in patients with mild-to-moderate (HR = 0.4, *P* < 0.001) and those with severe (HR = 0.4, *P* = 0.007) aGVHD. In addition, severe aGVHD (HR = 2.2, *P* < 0.001), the use of TBI in the conditioning regimen (HR = 1.7, *P* = 0.025), and sex mismatch (HR = 1.7, *P* = 0.003) were associated with a higher NRM rate, whereas the use of a matched-related donor (HR = 0.6, *P* = 0.004) and a lower dose of steroids at initiation of systemic therapy (<2 mg/kg, HR = 0.4, *P* = 0.02) were associated with a lower NRM rate. Hyperacute GVHD was associated with a significantly higher NRM in patients with severe aGVHD (HR = 2.05, *P* = 0.04), but not in patients with mild aGVHD (HR = 1.2, *P* = 0.4, Table 5). Covariates considered in multivariate analysis included response to first-line therapy, time of onset, and severity of aGVHD (severe hyperacute aGVHD, and severe aGVHD diagnosed after day 14), the use of TBI in the conditioning regimen, the use of a matched-related donor, and sex mismatch. Although the dose of steroid was significant on univariate analysis, it was not considered in multivariate analysis because only one of the 24 patients who received lower doses had severe aGVHD, which precludes adjusting for confounding variables. Results of the multivariate analysis were consistent with those of the univariate analysis and confirmed the independent prognostic value of the response to first-line therapy. 

We performed additional subanalyses to further demonstrate the independent effect of response on NRM by grouping patients based on the severity, timing of onset, and response of aGVHD (Table 6). Our data showed that response to therapy was associated with more favorable outcome in patients with mild-moderate aGVHD irrespective of time of onset. Similarly it was associated with lower NRM in patients with severe aGVHD diagnosed after day 14 after transplant. We could not evaluate the impact of response in patients with severe hyperacute GVHD because of sample size limitations. These findings indicate that response is an independent factor that adds prognostic value beyond the severity and timing of onset of aGVHD. 

## 4. Discussion

In this study, we evaluated patient, transplant, and aGVHD factors as predictors of response to standard first-line therapy. The purpose of this evaluation was to identify a subset of patients unlikely to receive significant benefit from corticosteroids and who may benefit from alternative therapeutic strategies. 

None of the patient demographic or transplant characteristics independently predicted response to corticosteroids. This highlights the need for the development of biomarker panels in the evaluation of aGVHD using a newer available technology [14]. 

Acute GVHD characteristics were the only factors significantly associated with response to first-line therapy, including the time of onset of aGVHD and its severity at the initiation of therapy. Indeed, in this series, only 15% of patients with severe, hyperacute GVHD had a CR or PR after initial therapy with corticosteroids. Patients with mild-to-moderate hyperacute aGVHD and severe nonhyperacute GVHD fared significantly better. 

We have previously reported that hyperacute GVHD occurs in 27% of grade II–IV aGVHD patients and is associated with a lower response rate to therapy and higher NRM [8]. Hyperacute GVHD may be related to damage induced by the conditioning regimen and its resulting inflammatory cytokine production. This pretransplantation physical damage may be less responsive to corticosteroids than typical GVHD, prompting a more durable cytokine production and thus a worse outcome. Severe aGVHD was also significantly associated with a worse response to therapy, consistent with previous studies [2, 15, 16]. 

Sex mismatch (female donor to male recipient) was associated with a decreased response to first-line therapy in severe aGVHD. It was also associated with higher NRM, independent of the severity and time of onset of aGVHD. Sex mismatch may contribute to an increased rate of aGVHD due to exposure of a parous female donor to non-self-antigens during pregnancy, thus priming the future donor immune system to recognize and attack host antigens. It is unclear why the mismatch would increase the risk for refractory aGVHD. 

Our data showed that day-14 response to first-line therapy provides additional independent prognostic value beyond severity and timing of aGVHD. In this study, response was associated with lower NRM in patients with mild-moderate aGVHD irrespective of the time of onset. It was also associated with lower NRM in severe aGVHD diagnosed after day 14 posttransplantation. We could not evaluate its impact in severe hyperacute GVHD because of sample size limitations. Recent publications have evaluated the validity of using response to therapy as primary endpoint in upfront aGVHD treatment trials [5–7, 14]. Although these reports found response at day 28 to be statistically a stronger predictor, both days 14 and 28 were associated with long-term outcomes and in good agreement. Our data compliment findings of these studies indicating that patients who do not respond to therapy by day 14 are likely to achieve inferior outcomes. In the current study, we could not compare response on day 14 with response assessed on days 28 or 56 due to the unavailability of consistent data on response to therapy at these time points. 

While response on day 28 is a valid endpoint that facilitates comparison of outcomes across clinical trials in aGVHD, it does not necessarily dictate the best clinical practice to improve outcomes in nonresponding patients. Early identification of these patients may allow for aggressive intervention and perhaps improve outcomes. Independent of the definition of steroid refractory aGVHD and the timing for second-line therapy used by each transplant center, rethinking the management of these patients prior to day 28 may be warranted. 

Inclusion of severity, time of onset, and day 14 response to first-line therapy in scoring criteria may aid in the early identification of patients who are unlikely to receive significant benefit from corticosteroids. These patients would, therefore, be eligible for investigational approaches, including customization of therapy and inclusion in clinical studies which consider more aggressive or innovative strategies earlier in the disease course [17]. Sex mismatch, the use of an unrelated donor, and the use of TBI in the conditioning regimen independently contribute to the mortality rate associated with aGVHD. Development of a scoring system that integrates these factors along with timing and severity of aGVHD, as well as early response is warranted in a larger study population. 

In conclusion, our results indicate that patients with severe hyperacute GVHD have inferior response to initial therapy and higher NRM rates. This patient subgroup is potentially eligible for innovative strategies that ideally could be implemented upfront, and not after steroid failure has occurred. Our results also suggest that a change in therapy is warranted by 2 weeks of corticosteroids in patients who have not developed at least a partial response.

## Figures and Tables

**Table 1:** Patients and transplant characteristics.

Characteristic	Value (%)	
No. of patients	287	
*Patient age *		
Median, y (range)	45	(19–72)
Older than 40 y	138	(48)
40 y or younger	149	(52)
Median no. of prior chemotherapy lines (range)	2	(0–9)

*Donor/patient sex mismatch*		
Female Donor/Male Patient	73	(25)
Other	214	(75)

*Diagnosis *		
Lymphoid	163	(57)
Myeloid	100	(35)
Multiple myeloma	8	(3)
Solid tumor	4	(1)
Other	12	(4)

*Disease status at transplantation *		
Not in remission	188	(66)
Remission	99	(34)

*Donor type *		
Matched	251	(88)
Mismatched	36	(12)

*Cell source *		
Peripheral Blood	126	(44)
Bone Marrow	161	(56)

*Conditioning regimen *		
Reduced intensity	102	(35.5)
High-dose chemotherapy	137	(50.5)
High-dose chemotherapy/TBI	48	(13.9)

**Table 2:** Evaluation of predicators of first-line therapy for treatment of acute GVHD.

Predictor	No. of patients	CR/PR, no. of patients	OR	Univariate 95% CI	*P*
Grade at first line therapy					
I	89	58 (65)	Reference	—	—
II	141	81 (57)	0.7	0.4–1.2	0.2
III	54	21 (39)	0.3	0.2–0.7	0.003
IV	3	1 (33)	0.3	0.02–3.1	0.3
Grade III-IV			0.4	0.2–0.7	0.003

Age					
≤45 (median)	138	74 (54)	Reference	—	—
>45	149	87 (58)	1.2	0.8–1.9	0.4

Gender					
F	112	60 (54)	0.8	0.5–1.4	0.5
M	175	101 (58)	Reference	—	—

Donor/patient sex					
Female/Male	73	37 (51)	0.7	0.4–1.3	0.3
Other	214	124 (58)	Reference	—	—

Conditioning regimen					
Total body irradiation	48	27 (56)	1.1	0.5–2.2	0.8
High Dose, No total body irradiation	137	90 (57)	1.1	0.6–1.9	0.7
Reduced intensity	102	44 (54)	Reference		

Allotype					
Matched	251	146 (58)	1.9	0.96–3.9	0.06
Mismatched	36	15 (42)	Reference	—	—

Cell type					
BM	161	87 (54)	0.8	0.5–1.3	0.4
PB	126	74 (59)	Reference	—	—

Diagnosis					
Myeloid	163	90 (55)	0.6	0.2–2.0	0.4
Lymphoid	100	59 (59)	Reference	—	—
Multiple myeloma	8	3 (37)	Excluded	—	—
Other	4	4 (100)	Excluded	—	—
Solid	12	5 (42)	Excluded	—	—

Disease status at transplant					
Active disease	188	113 (60)	1.6	0.98–2.6	0.06
No active disease	99	48 (48)	Reference	—	—

Prior chemotherapy regimens					
≤2	182	100 (55)	Reference	—	—
>2	105	61 (58)	1.1	0.7–1.8	0.6

Dose of methylprednisolone first line					
<2 mg	24	15 (62)	Reference	—	—
2 mg	250	144 (58)	0.8	0.3–1.9	0.6
Unknown	13	2 (15)	Excluded		

Days from transplant to aGVHD					
≤14	73	31 (42)	Reference	—	—
>14	214	130 (61)	0.5	0.3–0.8	0.007

CD34 infused (quartile)					
≤3.5	72	40 (56)	Reference	—	—
>3.5 to ≤4.3	68	42 (62)	1.3	0.7–2.5	0.5
>4.3 to ≤5.5	73	40 (55)	0.97	0.5–1.9	0.9
>5.5	70	37 (37)	0.9	0.5–1.7	0.7

**Table 3:** Effect of severity and time of onset of aGVHD on response.

	Total no. of patients *N* = 287 (%)	CR/PR, no. of patients (%)	OR	95% CI	*P*
Grade I-II and no Hyperacute GVHD	170 (59)	110 (65)	Reference	—	—
Grade I-II and Hyperacute GVHD	60 (21)	29 (48)	0.5	0.3–0.9	0.03
Grade III-IV and no Hyperacute GVHD	44 (15)	20 (45)	0.4	0.2–0.9	0.02
Grade III-IV and Hyperacute GVHD	13 (5)	2 (15)	0.1	0.02–0.5	0.003

**Table 4:** Primary cause of death among patients with aGVHD.

Causes of death	CR/PR	Refractory
	no. of deaths (%)	no. of deaths (%)
	115	109
Acute GVHD	4 (3)	38 (35)
Chronic GVHD	30 (26)	32 (29)
Disease persistence/recurrence	49 (43)	23 (21)
Infections	13 (11)	9 (8)
Other	15 (13)	6 (5)
Unknown	4 (3)	1 (1)

**Table 5:** Univariate predictors of nonrelapse morality among patients with acute GVHD at 2 years.

Predictor	*N* = 287	HR	95% CI	*P*
Age (quartile)				
≤35	69	Reference	—	—
>35 to ≤45	69	1	0.7–1.7	0.8
>45 to ≤55	83	0.9	0.6–1.5	0.8
>55	66	1.3	0.8–2.1	0.3

Dose of first line				
<2 mg	24	0.4	0.2–0.9	0.02
2 mg	250	Reference	—	—
Unknown	13	Reference	—	—

Response to first line				
CR/PR	161	0.4	0.3–0.5	<0.001
Other	126	Reference	—	—

Grade and hyper aGVHD				
Grade I-II, no hyperacute GVHD	170	Reference	—	—
Grade I-II, hyperacute GVHD	60	1.2	0.8–1.9	0.4
Grade III-IV, no hyperacute GVHD	44	1.9	1.25–3.0	0.003
Grade III-IV, hyperacute GVHD	13	4.3	2.3–7.9	<0.001

Sex mismatch				
Yes	73	1.7	1.2–2.4	0.003
No	214	Reference	—	—

Conditioning regimen				
Total body irradiation	48	1.7	1.1–2.8	0.025
High dose, No total Body irradiation	158	0.97	0.6–1.4	0.9
Reduced intensity	81	Reference	—	—

Allotype				
Matched-related	139	0.6	0.4–0.85	0.004
Matched unrelated	112	Reference	—	—
Antigen mismatch-related	36	Reference	—	—

Cell type				
Bone marrow	161	1.1	0.8–1.6	0.5
Peripheral blood	126	Reference	—	—

Diagnosis				
Myeloid	163	Reference	—	—
Lymphoid	100	0.9	0.7–1.4	0.9
Multiple myeloma	8	0.6	0.2–2	0.4
Other	4	0.6	0.1–2.5	0.5
Solid	12	0.6	0.2–1.7	0.3

Disease status at transplant				
Active disease	188	1.1	0.8–1.5	0.7
No active disease	99	Reference	—	—

Prior chemotherapy regimens				
≤2	182	Reference	—	—
>2	105	1.3	0.9–1.8	0.2

CD34 infused (10e6 cells, quartile)				
≤3.5	72	Reference	—	—
>3.5 to ≤4.3	68	0.9	0.6–1.4	0.6
>4.3 to ≤5.5	73	0.7	0.5–1.2	0.2
>5.5	70	1.1	0.7–1.8	0.6

**Table 6:** The Effect of severity, hyperacute GVHD, and response to corticosteroids on nonrelapse morality.

Hyperacute					NRM at 100 days			NRM at 1 year		
Group	Grade	GVHD	CR or PR	*N*	% CI	HR	*P*	% CI	HR	*P*
1	1 or 2	No	Yes	110	9%	Reference	—	27%	Reference	—
2	1 or 2	No	No	60	23%	2.8	0.01	55%	2.5	<0.001
3	1 or 2	Yes	Yes	29	3%	0.4	0.3	34%	1.1	0.7
4	1 or 2	Yes	No	31	31%	4.9	<0.001	45%	2.5	0.003
5	3 or 4	No	Yes	20	25%	2.9	0.05	35%	1.4	0.4
6	3 or 4	No	No	24	50%	7.6	<0.001	75%	4.7	<0.001
7	3 or 4	Yes	Yes	2	100%	Excluded	—	100%	Excluded	—
8	3 or 4	Yes	No	11	70%	11.6	<0.001	80%	6.2	<0.001

% CI: percent cumulative incidence; NRM: nonrelapse mortality; —: nonapplicable, KM: Kaplan-Meier.
